# The Prognostic Impact of Body Composition for Locally Advanced Breast Cancer Patients Who Received Neoadjuvant Chemotherapy

**DOI:** 10.3390/cancers13040608

**Published:** 2021-02-04

**Authors:** Toshiaki Iwase, Aaroh Parikh, Seyedeh S. Dibaj, Yu Shen, Tushaar Vishal Shrimanker, Sudpreeda Chainitikun, Kumiko Kida, Maryanne E. Sapon, Onur Sahin, Anjali James, Andrea Yizel Delgado Medrano, Ann H. Klopp, Naoto T. Ueno

**Affiliations:** 1 Section of Translational Breast Cancer Research, Department of Breast Medical Oncology, The University of Texas MD Anderson Cancer Center, 1515 Holcombe Boulevard, Unit 1354, Houston, TX 77030, USA; tiwase@mdanderson.org (T.I.); tushaar.shrimanker@greenwichhospital.org (T.V.S.); sudpreeda.cha@medparkhospital.com (S.C.); kidakumi@luke.ac.jp (K.K.); mesapon@mdanderson.org (M.E.S.); osahin1@mdanderson.org (O.S.); ajames2@mdanderson.org (A.J.); 2 Morgan Welch Inflammatory Breast Cancer Research Program and Clinic, The University of Texas MD Anderson Cancer Center, 1515 Holcombe Boulevard, Houston, TX 77030, USA; 3 Department of Radiation Oncology, The University of Texas MD Anderson Cancer Center, 1515 Holcombe Boulevard, Houston, TX 77030, USA; amparikh@bcm.edu (A.P.); aydelgado@mdanderson.org (A.Y.D.M.); aklopp@mdanderson.org (A.H.K.); 4 Department of Biostatistics, The University of Texas MD Anderson Cancer Center, 1515 Holcombe Boulevard, Houston, TX 77030, USA; sdibaj@mdanderson.org (S.S.D.); yshen@mdanderson.org (Y.S.)

**Keywords:** obesity, body composition, breast neoplasm, pathologic complete response, computed tomography

## Abstract

**Simple Summary:**

We aimed to determine the prognostic role of body composition in patients with breast cancer who received neoadjuvant chemotherapy. Previous studies suggested that body composition is a better indicator of breast cancer treatment outcome than body mass index. A comprehensive body composition analysis found that a low ratio of total visceral adipose tissue to subcutaneous adipose tissue was associated with shorter overall survival. This finding will lead to further investigation of the role of body composition in outcomes for patients with locally advanced breast cancer.

**Abstract:**

Our previous study indicated that a high amount of visceral adipose tissue was associated with poor survival outcomes in patients with early breast cancer who received neoadjuvant chemotherapy. However, inconsistency was observed in the prognostic role of body composition in breast cancer treatment outcomes. In the present study, we aimed to validate our previous research by performing a comprehensive body composition analysis in patients with a standardized clinical background. We included 198 patients with stage III breast cancer who underwent neoadjuvant chemotherapy between January 2007 and June 2015. The impact of body composition on pathologic complete response and survival outcomes was determined. Body composition measurements had no significant effect on pathologic complete response. Survival analysis showed a low ratio of total visceral adipose tissue to subcutaneous adipose tissue (V/S ratio ≤ 34) was associated with shorter overall survival. A changepoint method determined that a V/S ratio cutoff of 34 maximized the difference in overall survival. Our study indicated the prognostic effect of body composition measurements in patients with locally advanced breast cancer compared to those with early breast cancer. Further investigation will be needed to clarify the biological mechanism underlying the association of V/S ratio with prognosis in locally advanced breast cancer.

## 1. Introduction

Obesity is a major risk factor for metabolic dysfunction and is associated with poor treatment response and prognosis in breast cancer. A previous study showed that high body mass index (BMI) was associated with lower pathologic complete response (pCR) rates and shorter disease-free survival in patients with breast cancer who underwent neoadjuvant chemotherapy (NACT) [1,2,3].

Over the past decade, many studies have used BMI as a clinical indicator of obesity because of its simplicity and clinical availability. However, the prognostic value of BMI was sometimes inconsistent when used to evaluate breast cancer treatment outcomes [4,5,6]. Moreover, studies have shown that a certain percentage of patients with high BMI are metabolically healthy with adipose tissue’s normal function, which has been called the “obesity paradox” [7,8]. A plausible explanation for the obesity paradox is that BMI does not always reflect body composition, defined as the constitution of fat, muscle mass, bone, and water in the body. Understanding body composition is important for breast cancer treatment because recent studies indicated that body composition could be an imaging biomarker for insulin resistance, response to treatment, and prognosis in patients with breast cancer [9,10,11].

We previously reported that patients with early breast cancer who had a higher amount of visceral adipose tissue (VAT) and the lower quality of VAT represented by the Hounsfield unit (HU) measured by computed tomography in the upper abdominal area had significantly shorter distant disease-free survival than those with a lower amount and higher quality of VAT, owing to increasing insulin resistance [9,10]. In addition to VAT, a significantly reduced amount of skeletal muscle mass (i.e., sarcopenia) was also associated with higher overall mortality rates in breast cancer survivors [12]. Moreover, the coexistence of obesity and sarcopenia, called sarcopenic obesity, creates a vicious cycle of chronic inflammation and insulin resistance, leading to breast cancer progression [11,13,14].

Given these study results, applying body composition as a prognostic imaging biomarker would be feasible in breast cancer treatment. However, many of the previous studies have focused on early-stage breast cancer or unstandardized stage of breast cancer, and little is known about the prognostic value of body composition in locally advanced breast cancer. In contrast to early-stage breast cancer, a recent study showed that subcutaneous adipose tissue (SAT) played a prognostic role in patients with locally advanced breast cancer, and increased VAT was associated with a lower risk of death [15]. These inconsistent results indicate that body composition plays different roles at different clinical stages of breast cancer.

To clarify the role of body composition in breast cancer treatment, we performed a comprehensive body composition analysis by measuring the total amount of VAT (tVAT), the total amount of SAT (tSAT), VAT-HU, skeletal muscle index (SMI), and tVAT to tSAT ratio (V/S ratio) in patients with breast cancer who underwent NACT, to validate our previous research in patients with a standardized clinical background. We found that a low ratio of V/S ratio was associated with shorter overall survival (OS). In contrast, well-known body composition measurements related to pCR and survival outcomes were not associated with either in our patient population. This new insight indicates that body composition measurements have a different prognostic value between early and locally advanced breast cancer, and further research on the biology of body composition is needed.

## 2. Results

### 2.1. Patient Demographics and Baseline Characteristics

Table 1 shows the demographic and baseline characteristics of the 198 patients included in our analysis. Forty-six patients (23%) achieved pCR. The median age was 49 years, and most patients (112, 57%) were premenopausal. More than half of the patients were white (54%), 21% were black, and 19% were Spanish/Hispanic. Fifty-eight percent of the patients had estrogen receptor (ER)+/human epidermal growth factor receptor 2 (HER2)−disease. Rates of ER+/HER2+, ER−/HER2+, and triple-negative breast cancer were 17%, 8%, and 17%, respectively. All patients had stage III disease, and 47 patients (24%) had inflammatory breast cancer. Sixty-eight percent of patients had T3 or T4 disease, and almost half of the patients had N3 disease (48%).

### 2.2. Correlation between BMI and Body Composition

BMI was significantly correlated with tVAT, VAT-HU, tSAT, V/S ratio, and SMI. A significant positive correlation was observed with tVAT and tSAT (Spearman R = 0.77 and 0.89, respectively, *p* < 0.0001 for both), and a significant negative correlation was observed with VAT-HU and SMI (Spearman R = −0.53 and −0.63, respectively, *p* < 0.0001 for both). The correlation between BMI and V/S ratio had marginal significance (Spearman R = 0.14, *p* = 0.042).

### 2.3. Univariate Analysis for the Effect of Body Composition Measurements for pCR

With the primary outcome of pCR, a univariate logistic regression model was conducted using the covariates of age, race, BMI, tVAT, VAT-HU, tSAT, subtype, and presence of inflammatory breast cancer. The model showed that only subtype was associated with pCR (*p* < 0.0001). BMI (*p* = 0.41) and body composition measurements including tVAT (*p* = 0.62), VAT-HU (*p* = 0.56), and tSAT (*p* = 0.19) did not show any significant effect for pCR.

### 2.4. Survival Analysis

The median follow-up time for all 198 patients was 4.7 years. A proportional hazards assumption indicated that African American race, triple-negative breast cancer, and low V/S ratio were associated with shorter OS (Table 2). BMI (*p* = 0.66 for obese vs. underweight/normal and *p* = 0.34 for overweight vs. underweight/normal) and body composition measurements including tVAT (*p* = 0.31), VAT-HU (*p* = 0.60), tSAT (*p* = 0.78), and SMI (*p* = 0.83) did not show any significant prognostic value in the model.

**Figure 1:**
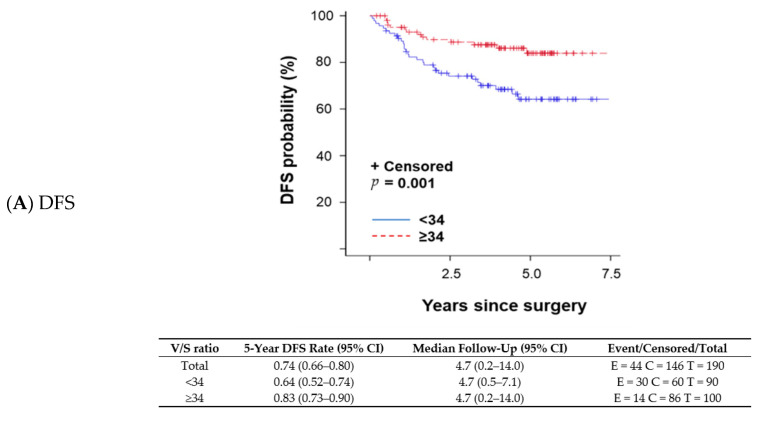

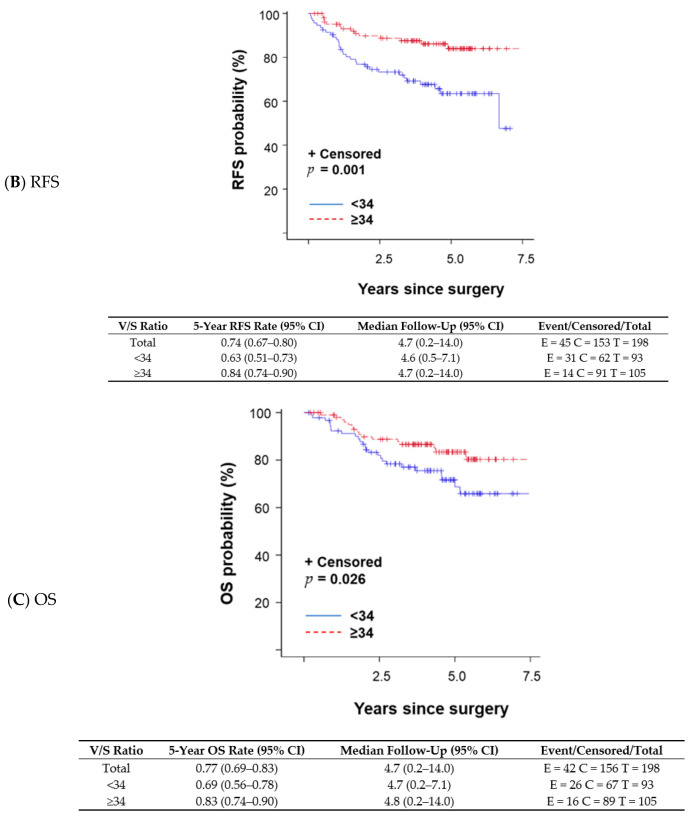
Kaplan–Meier survival plots and detailed survival rates stratified by the ratio of total visceral adipose tissue to total subcutaneous adipose tissue (V/S ratio) for (**A**) distant recurrence-free survival (DFS), (**B**) any recurrence-free survival (RFS), and (**C**) overall survival (OS). Abbreviations: CI, confidence interval.

A changepoint method based on the log-rank test determined that V/S ratio = 34 was the best cutoff to discriminate survival outcomes; patients with a V/S ratio < 34 had shorter OS than those with a V/S ratio ≥ 34. The V/S ratio cutoff value of 34 was also associated with distant recurrence-free survival (DFS; *p* = 0.001 per log-rank test; Figure 1A), any recurrence-free survival (RFS; *p* < 0.001 per log-rank test; Figure 1B), and OS (*p* = 0.026 per log-rank test; Figure 1C). A univariate proportional hazards regression model indicated that patients with a V/S ratio < 34 had a higher risk of an event (distant recurrence, any recurrence, or death) anytime during their follow-up period compared with those with a V/S ratio ≥ 34. OS was not significantly different between patients with sarcopenic obesity and those without (*p* = 0.824 per log-rank test). We fit a multivariable model to assess V/S ratio while adjusting subtype (TNBC vs. other subtypes) in the Cox model (*p* = 0.004). OS remained the same trend that patients with V/S ratio < 34 had worse OS (OS; *p* = 0.102) with a marginal significance.

## 3. Discussion

Our comprehensive imaging analysis showed that a low V/S ratio is associated with poor survival outcomes in patients with locally advanced breast cancer who have received NACT. However, previously reported prognostic body composition measurements, including tVAT, tSAT, and SMI did not have any significant effect on pCR or survival outcomes.

A low V/S ratio could be interpreted in two ways: (1) low tVAT relative to tSAT or (2) high tSAT relative to tVAT. However, how these conditions contribute to poor survival outcomes for patients with locally advanced breast cancer is not clearly explained in previous reports. For VAT, many previous preclinical and clinical studies have reported that abdominal VAT had a vital role in promoting breast cancer progression. A gene expression analysis of 17 healthy women showed that VAT has a distinct metabolic function that can produce pro-inflammatory cytokines, including CC chemokine receptor 2 and macrophage migration inhibitory factor, and this potentially leads to breast cancer progression [16]. Clinically, increased VAT is associated with insulin resistance because VAT induces free fatty acid accumulation in the liver via portal vein circulation [17]. Our previous study showed that increased amount and lower quality of VAT was associated with shorter distant disease-free survival via increasing insulin resistance [9]. Many other preclinical and clinical studies have reported a positive relationship between increased VAT, poor NACT outcome [18], and shorter survival outcome [9,10]; however, recent studies have indicated that deep abdominal SAT (daSAT)—defined as adipose tissue at the deepest layer of abdominal SAT—is also metabolically active and has a similar metabolic function to that of VAT. A clinical study showed that the depth of daSAT was a strong predictor of insulin resistance [19], as well as increased expression of pro-inflammatory, lipogenic, and lipolytic genes [20]. An imaging analysis with proton magnetic resonance spectroscopy showed that daSAT was more saturated than the superficial SAT, which could be attributed to a high ratio of saturated to monounsaturated fatty acids [21]. Thus, the SAT’s metabolic function varies according to its anatomic location, and the function of daSAT is similar to that of VAT. Because the present study did not anatomically discriminate daSAT from tSAT, how daSAT affected SAT’s prognostic value could not be determined. In future studies, investigating the metabolic mechanism of daSAT will be crucial to elucidate its prognostic value in the treatment of locally advanced breast cancer.

The other body composition measurement we investigated, SMI, did not show any significant prognostic value in the present study. Moreover, in the exploratory analysis, sarcopenic obesity was not a prognostic factor, inconsistent with previous studies [11,22]. The prognostic role of SMI in the treatment of breast cancer has been mainly discussed in the context of sarcopenia, and recently the concept of sarcopenic obesity emerged as a new prognostic indicator for patients with breast cancer. Patients with non-metastatic breast cancer who had sarcopenia had a higher overall mortality rate than those without sarcopenia [22]. In that study, the highest mortality rate was observed in patients with sarcopenia and high total adipose tissue [22]. The present study defined sarcopenic obesity by combining BMI with SMI and did not consider the quality of skeletal muscle or actual muscle function. Skeletal muscle is a key component of body composition in breast cancer because a decrease in skeletal muscle induces chronic inflammation and insulin resistance [13,14]. To obtain a definitive conclusion for the prognostic role of SMI, future studies must integrate quality and functional data for skeletal muscle with survival analysis, as well as validate the cutoff for SMI in a large cohort.

The present study was unique in that we included only patients with locally advanced breast cancer (stage III) treated with NACT. Many body composition studies in breast cancer have been performed using a diverse patient population, including mixed clinical stages and different treatment approaches. In addition, some studies had indefinite inclusion criteria and small sample size. The present study aimed to investigate patients with breast cancer who had a standardized clinical background because previous studies indicated no definitive conclusion could be obtained without standardizing the patient background. One previous body composition study showed that the prognostic value of body composition measurements varied according to clinical stage. A body composition analysis in 3235 women showed that although VAT had a positive relationship with mortality rates in patients with stage II breast cancer, an inverse relationship was observed in those with stage III breast cancer [15]. Moreover, SAT was more prognostic than VAT in patients with stage III breast cancer [15]. This result partly supported our finding that patients with locally advanced breast cancer who had a high V/S ratio had better survival outcomes. Because the effect of obesity on metabolic change in adipose tissue, as well as survival outcomes, is chronic and moderate compared with the other clinical prognostic factors, the prognostic role of body composition measurements in patients with locally advanced breast cancer might be different from the prognostic role of these measurements in patients with early-stage breast cancer. Future detailed studies must include a comprehensive metabolic analysis to conclusively determine the role of body composition measurements in locally advanced breast cancer.

The present study has some limitations. First, we excluded a number of patients during the screening process. This procedure could have resulted in selection bias. Second, our imaging analysis did not cover the pelvic area, and the adipose tissue from the umbilicus level to the end of the pelvis was missed in the analysis. Because differences in body shape affect the location of fat deposits, these missing values may have affected the imaging analysis results which was discrepant from the previous report. Third, the number of included cases was small because of the detailed imaging analysis. The modest sample size and small number of deaths limited our ability to fit a multivariable model adjusting multiple baseline covariates. It is worth noting a modest correlation between V/S ratio and subtype may lead to a less significant association between V/S ratio and OS in the multivariable model given the limited number of death events. Furthermore, we included 24% of IBC patients in the present study, which may not represent a fraction of common stage III breast cancer patients.

In summary, the present study showed that the decreased V/S ratio was associated with a poor survival outcome for the patient with locally advanced breast cancer. In contrast, well-known body composition measurements related to pCR and survival did not show any significant values. Our result indicated the prognostic effect of body composition measurements in the patient with locally advanced breast cancer is different from early breast cancer.

## 4. Materials and Methods

### 4.1. Materials

We screened patients with breast cancer who underwent NACT between January 2007 and June 2015 using the following inclusion criteria: (1) pathologically confirmed stage III invasive breast cancer per American Joint Committee on Cancer diagnostic criteria; (2) underwent multi-detector computed tomography with images spanning from the top of the diaphragm to the umbilicus level before initiating NACT; (3) thickness of the CT slices was at least 5 mm; and (4) sufficient clinical and pathologic information and follow-up data for analysis. In the patient selection process, initial screening detected 266 potentially eligible patients. Among these patients, 7 were unable to undergo imaging analysis, 52 had missing clinical and pathologic information, and 9 had missing follow-up information. As a result, a total of 198 patients were included in the final analysis. We used stocked Digital Imaging and Communications in Medicine (DICOM) data in the picture archiving and communication system at The University of Texas MD Anderson Cancer Center for imaging analysis. Clinical and pathologic information was extracted from the electronic health records system at MD Anderson.

### 4.2. Body Composition Measurement

Body composition analysis included tVAT (cm^3^), tSAT (cm^3^), the average CT HU value for tVAT and tSAT, and SMI. The area used for the calculation of tVAT and tSAT was designated as that spanning from the top of the diaphragm to the navel level in the CT axial view. We also measured the V/S ratio to evaluate how the balance of tVAT and tSAT affected the outcomes. The sum of the skeletal muscle area at the third lumbar level was normalized by dividing the body surface area and defined as SMI (cm^2^/m^2^). This process was necessary to accurately determine the muscle mass volume, which was greatly affected by body shape. tVAT and tSAT were divided by the median value for the univariate logistic regression model and proportional hazards assumption. As an exploratory outcome measure, we also evaluated the prognostic impact of sarcopenic obesity. Sarcopenic obesity was defined as BMI ≥ 30 and SMI lower than the median value.

Three-dimensional imaging analysis for adipose tissue was performed using in-house software, Medical Executable for the Efficient and Robust Quantification of Adipose Tissue (MEERQAT, Department of Radiology, MD Anderson), which was developed using MATLAB software (Mathworks, Natick, MA, USA). MEERQAT is 3-dimensional (3D) imaging analysis software that distinguishes VAT from SAT by manually identifying the region of interest. The actual imaging process of MEERQAT was previously described by Parikh et al. [23]. Briefly, CT images were transferred into MEERQAT program as a series of DICOM images and reconstructed into a 3D volume and displayed in the axial, coronal, and sagittal view. The upper (top of the diaphragm) and lower boundaries (navel level) were manually defined to analyze the region of interest (ROI). After defining the boundaries, VAT and SAT were separated by drawing elliptical contours. The ellipses were shaped inside the rectus abdominis and transverse abdominis extending posteriorly to the vertebral body so that VAT was within the ellipse and SAT was outside. The ellipses were drawn on the upper, middle, and lower quartiles, and the top and bottom slices of the ROI. During this process, the optimal location and size of the ellipse to separate VAT and SAT were determined. The program then linearly interpolated these contours for the remainder of the slices, separating the 3D ROI into two regions: the area enclosed by the ellipse contained the abdominal cavity, which included VAT and organs; the area outside of the ellipse contained SAT, skin, and air outside of the patient. Finally, the program calculated total adipose tissue volume by automatically counting the number of voxels between −190 and −30 HU and multiplying this total by the volume of each voxel.

Two-dimensional imaging analysis for skeletal muscle was performed using NIH Image J (NIH, Bethesda, MA, USA). The HU range for detecting muscle mass was selected as −29 to 150 HU based on a previous report [24].

### 4.3. Outcomes

The pre-specified primary outcome of the study was pCR, confirmed by definitive surgery after NACT. pCR was defined as either an absence of residual tumor (ypT0N0) or noninvasive in situ residual tumor remaining (ypTis/0N0) in the surgical specimen from the primary tumor and axillary lymph nodes. Pathologic evaluation was performed by pathologists at MD Anderson. Secondary outcomes included DFS, RFS, and OS. DFS was defined as the time from the initial diagnosis to recurrence in distant organs, including bone, liver, lung, or brain. RFS was defined as the time from the initial diagnosis to recurrence in any site, including locoregional recurrence or recurrence in the contralateral breast. OS was defined as the time from the initial diagnosis to death from any cause. A data manager at MD Anderson performed a follow-up survey by contacting patients by mail up to 10 years after the patient was referred to community health care providers. Patients who were lost to follow-up were censored.

### 4.4. Statistical Considerations

Data were first summarized by descriptive statistics such as mean, standard deviation, median, and range for continuous variables and frequency and proportion for categorical variables. A Spearman correlation coefficient was calculated to evaluate the relationship between BMI, age, and body composition measurements. A univariate logistic regression model was fitted to evaluate the association between clinical characteristics and the probability of pCR. A changepoint method based on a log-rank test was applied to find a cutoff for the V/S ratio [25]. Kaplan–Meier estimates of DFS, RFS, and OS were calculated and plotted as a function of time. Univariate proportional hazards regression models were fitted to further evaluate these associations. The proportional hazards assumption was checked for each of the covariates. We considered two-sided *p* < 0.05 to be statistically significant. All statistical analysis was performed using R software (R Foundation).

## 5. Conclusions

The present study showed that a low V/S ratio was associated with poor survival outcomes and the previously reported tVAT variable had no significant effect on pCR and survival. Further investigation will be needed to clarify the biological mechanism underlying the association of V/S ratio with prognosis in locally advanced breast cancer.

## Figures and Tables

**Table 1:** Patient demographic and baseline characteristics (*n* = 198).

Variable	WHO BMI Classification, No. (%)			Overall Cohort, No. (%)
	Underweight/Normal, 18.5 ≤ BMI < 25	Overweight, 25 ≤ BMI < 30	Obese, BMI ≥ 30	
No. of patients	63 (32)	62 (31)	73 (37)	
Age				
Median (Min, Max)	48 years (22, 73)	48.5 years (25, 72)	51 years (24, 80)	49 years (22, 80)
Race				
White	39 (36)	34 (32)	34 (32)	107 (100)
African American	8 (20)	16 (39)	17 (41)	41 (100)
Spanish/Hispanic	9 (24)	8 (21)	21 (55)	38 (100)
Asian	7 (58)	4 (33)	1 (8)	12 (100)
T category				
1	6 (67)	3 (33)	0 (0)	9 (100)
2	13 (24)	18 (33)	23 (43)	54 (100)
3	33 (38)	19 (22)	35 (40)	87 (100)
4	11 (23)	22 (46)	15 (31)	48 (100)
N category				
0	2 (18)	5 (46)	4 (36)	11 (100)
1	19 (31)	17 (28)	25 (41)	61 (100)
2	12 (39)	8 (26)	11 (35)	31 (100)
3	30 (31)	32 (34)	33 (35)	95 (100)
Histologic grade				
1	4 (40)	3 (30)	3 (30)	10 (100)
2	13 (20)	28 (43)	24 (37)	65 (100)
3	46 (37)	31 (26)	46 (37)	123 (100)
Reproductive status				
Premenopausal	42 (38)	36 (32)	34 (30)	112 (100)
Menopausal	21 (25)	26 (30)	39 (45)	86 (100)
ER				
Negative	16 (32)	12 (24)	22 (44)	50 (100)
Positive	47 (32)	50 (34)	51 (34)	148 (100)
PR				
Negative	29 (35)	25 (30)	29 (35)	83 (100)
Positive	34 (30)	37 (32)	44 (38)	115 (100)
HER2				
Negative	45 (30)	45 (30)	58 (40)	148 (100)
Positive	18 (36)	17 (34)	15 (30)	50 (100)
Subtype				
ER+/HER2−	36 (32)	36 (32)	42 (36)	114 (100)
ER+/HER2+	11 (33)	14 (41)	9 (26)	34 (100)
TNBC	9 (26)	9 (26)	16 (48)	34 (100)
ER−/HER2+	7 (44)	3 (19)	6 (37)	16 (100)
pCR				
non-pCR	47 (31)	48 (32)	57 (37)	152 (100)
pCR	16 (35)	14 (30)	16 (35)	46 (100)
IBC				
No	52 (35)	41 (27)	58 (38)	151 (100)
Yes	11 (23)	21 (45)	15 (32)	47 (100)

Abbreviations: WHO, World Health Organization; BMI, body mass index; ER, estrogen receptor; PR, progesterone receptor; HER2, human epidermal growth factor receptor 2; TNBC, triple-negative breast cancer; pCR, pathologic complete response; IBC, inflammatory breast cancer.

**Table 2:** Proportional hazards assumption for overall survival.

Variable	Reference	Hazard Ratio (95% CI)	*p*
Age *		0.99 (0.97–1.02)	0.53
Race			
Asian	White	0.90 (0.21–3.89)	0.89
African American	White	2.61 (1.31–5.20)	0.01
Spanish/Hispanic	White	0.97 (0.41–2.33)	0.95
BMI			
Obese	Underweight/normal	1.19 (0.55–2.59)	0.66
Overweight	Underweight/normal	1.46 (0.68–3.15)	0.34
tVAT			
High tVAT ^†^	Low tVAT	0.73 (0.39–1.35)	0.31
tSAT			
High tSAT ^†^	Low tSAT	1.09 (0.60–2.00)	0.78
V/S ratio			
V/S ratio < 34	V/S ratio ≥ 34	2.00 (1.07–3.74)	0.03
VAT-HU *		1.00 (0.99–1.02)	0.60
SMI			
High SMI ^†^	Low SMI	1.07 (0.58–1.97)	0.83
Subtype			
ER+/HER2+	TNBC	0.04 (0.01–0.32)	0.002
ER+/HER2−	TNBC	0.39 (0.20–0.74)	0.004
ER−/HER2+	TNBC	0.23 (0.05–0.99)	0.048
IBC			
IBC	non-IBC	1.69 (0.86–3.30)	0.13

Abbreviations: CI, confidence interval; BMI, body mass index; tVAT, total visceral adipose tissue; HU, Hounsfield units; tSAT, total subcutaneous adipose tissue; SMI, skeletal muscle index; V/S ratio, ratio of tVAT to tSAT; ER, estrogen receptor; HER2, human epidermal growth factor receptor 2; TNBC, triple-negative breast cancer; IBC, inflammatory breast cancer. * Continuous variable; ^†^ tVAT, tSAT, and SMI were divided by the median value.

## Data Availability

The data presented in this study may be available on request to the corresponding author.
